# Context-sensitive network analysis identifies food metabolites associated with Alzheimer’s disease: an exploratory study

**DOI:** 10.1186/s12920-018-0459-2

**Published:** 2019-01-31

**Authors:** Yang Chen, Rong Xu

**Affiliations:** Department of Population and Quantitative Health Science, School of Medicine, Case Western Reserve University, Cleveland, OH 44106 USA

**Keywords:** Food metabolite, Network analysis, Alzheimer’s disease, Disease prevention

## Abstract

**Background:**

Diet plays an important role in Alzheimer’s disease (AD) initiation, progression and outcomes. Previous studies have shown individual food-derived substances may have neuroprotective or neurotoxic effects. However, few works systematically investigate the role of food and food-derived metabolites on the development and progression of AD.

**Methods:**

In this study, we systematically investigated 7569 metabolites and identified AD-associated food metabolites using a novel network-based approach. We constructed a context-sensitive network to integrate heterogeneous chemical and genetic data, and to model context-specific inter-relationships among foods, metabolites, human genes and AD.

**Results:**

Our metabolite prioritization algorithm ranked 59 known AD-associated food metabolites within top 4.9%, which is significantly higher than random expectation. Interestingly, a few top-ranked food metabolites were specifically enriched in herbs and spices. Pathway enrichment analysis shows that these top-ranked herb-and-spice metabolites share many common pathways with AD, including the amyloid processing pathway, which is considered as a hallmark in AD-affected brains and has pathological roles in AD development.

**Conclusions:**

Our study represents the first unbiased systems approach to characterizing the effects of food and food-derived metabolites in AD pathogenesis. Our ranking approach prioritizes the known AD-associated food metabolites, and identifies interesting relationships between AD and the food group “herbs and spices”. Overall, our study provides intriguing evidence for the role of diet, as an important environmental factor, in AD etiology.

## Background

Alzheimer’s disease (AD) is the sixth leading cause of death and affected 5.3 million people in 2015 in the United States [1]. Diet plays an important role in the disease development [2]. Epidemiological studies have shown that higher adherence to a Mediterranean-type diet is associated with lower risk for AD [3–5] and mild cognitive impairment [6, 7]. Evidence suggests that improper diet habits may accelerate the progression of neuron damage through increasing the concentration of pro-inflammatory mediators [8, 9]. In addition, a number of experimental studies have investigated individual food-derived substances, such as resveratrol [10], vitamin [11], and advanced glycation end products [12], and demonstrated their neuroprotective or neurotoxic effects. Systematic study of food metabolites and their associations with AD may offer insights into the disease-environment relationship and disease prevention, but currently remains unexplored.

**Fig. 1:**
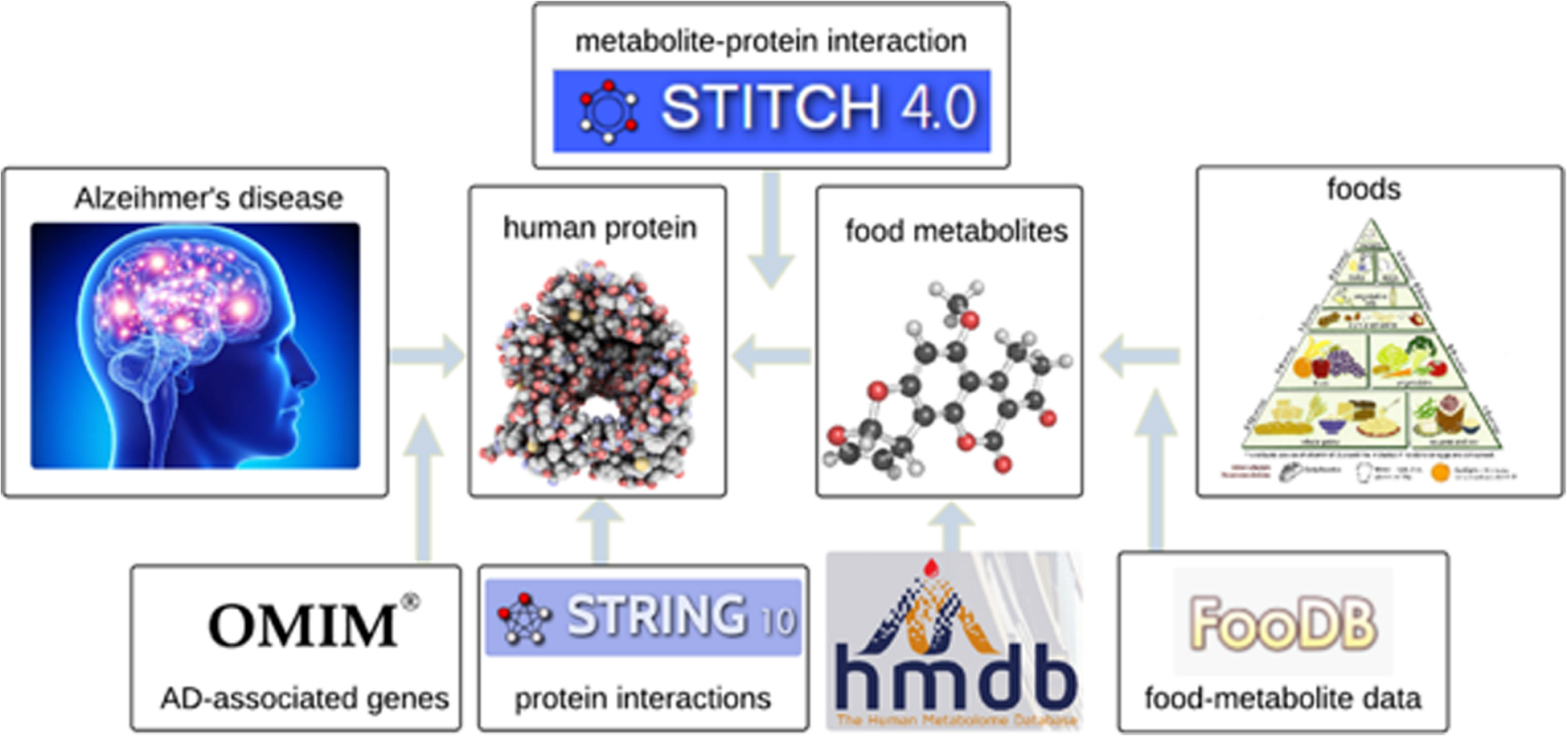
Link disease, chemical and genetic data to infer the food metabolites related with AD

Knowledge of metabolites and their interactions with disease-associated proteins has been obtained through in vitro, in silico, and in vivo technologies [13]. Most previous studies used these data to understand drug actions [14, 15]. Recently, large amounts of data have also accumulated on food metabolites (Fig. 1): The Human Metabolome Database (HMDB) [16] provides high-quality and comprehensive information for 74,462 metabolites, including their chemical, biological, and physical properties; these metabolites can be linked to foods using the large-scale food constitute resource in the Food Database (FooDB) [17], which covers the detailed compositional information for 907 foods. On the other hand, the interactions between the metabolites and human proteins are also available in chemical-protein interaction databases, such as the Search Tool for Interactions of Chemicals (STITCH) [18]. Here, we developed a network-based approach to integrate food metabolites with foods and human proteins, and performed a systematic unbiased study to identify AD-associated food metabolites.

Network-based approaches have been widely used in biomedical applications, such as predicting disease-gene associations [19–21], understanding disease comorbidity [22], and drug repurposing [23–25]. Traditional biomedical networks often model the relationships between two nodes based on pairwise similarities [26, 27]. For example, disease networks have been constructed by defining different similarities: some quantified the disease-disease similarities based on shared phenotypes [26, 27], and others used shared genetic factors [28]. These networks only captured the strength of the links, but ignored their semantic meaning. Real world interconnections are multi-typed. Specifically, in our problem, two metabolites may share commonalities because they are contained in the same food, or interact with the same protein. Recently, we introduce a novel concept—context-sensitive network [29], which preserves the context of how nodes are connected in the network. In a disease-gene prediction study, our experiment results demonstrated that the context-sensitive disease network led to significantly improved performance than the similarity-based disease network [29]. Analysis shows that the similarity-based network tends to contain noises and bury the true signals in a much denser network structure than the context-sensitive network [29]. Motivated by the benefits of context-sensitive networks, we construct a gene-metabolite-food (GMF) network in this study to model the complex relationships among food, metabolites, human proteins, and AD by seamlessly integrating heterogeneous databases in Fig. 1. Then we predict the food metabolites that are highly associated with AD using this network, and further investigate the pathways shared between AD and the prioritized food metabolites. Due to the lack of gold standard, we tested our approach in AD by manually curating a list of known AD-associated food metabolites. To the best of our knowledge, our study represents the first effort to systematically model the context-sensitive interactions among tens of thousands of human genes, food metabolites, food and diseases and to understand which and how food and food-derived metabolites are involved in disease development. In summary, the identification of food and food-derived metabolites and the understanding of their role as key mediators through which these factors promote or protect against human diseases will enable new possibilities for disease understanding, diagnosis, prevention, and treatment.

## Methods

**Fig. 2:**
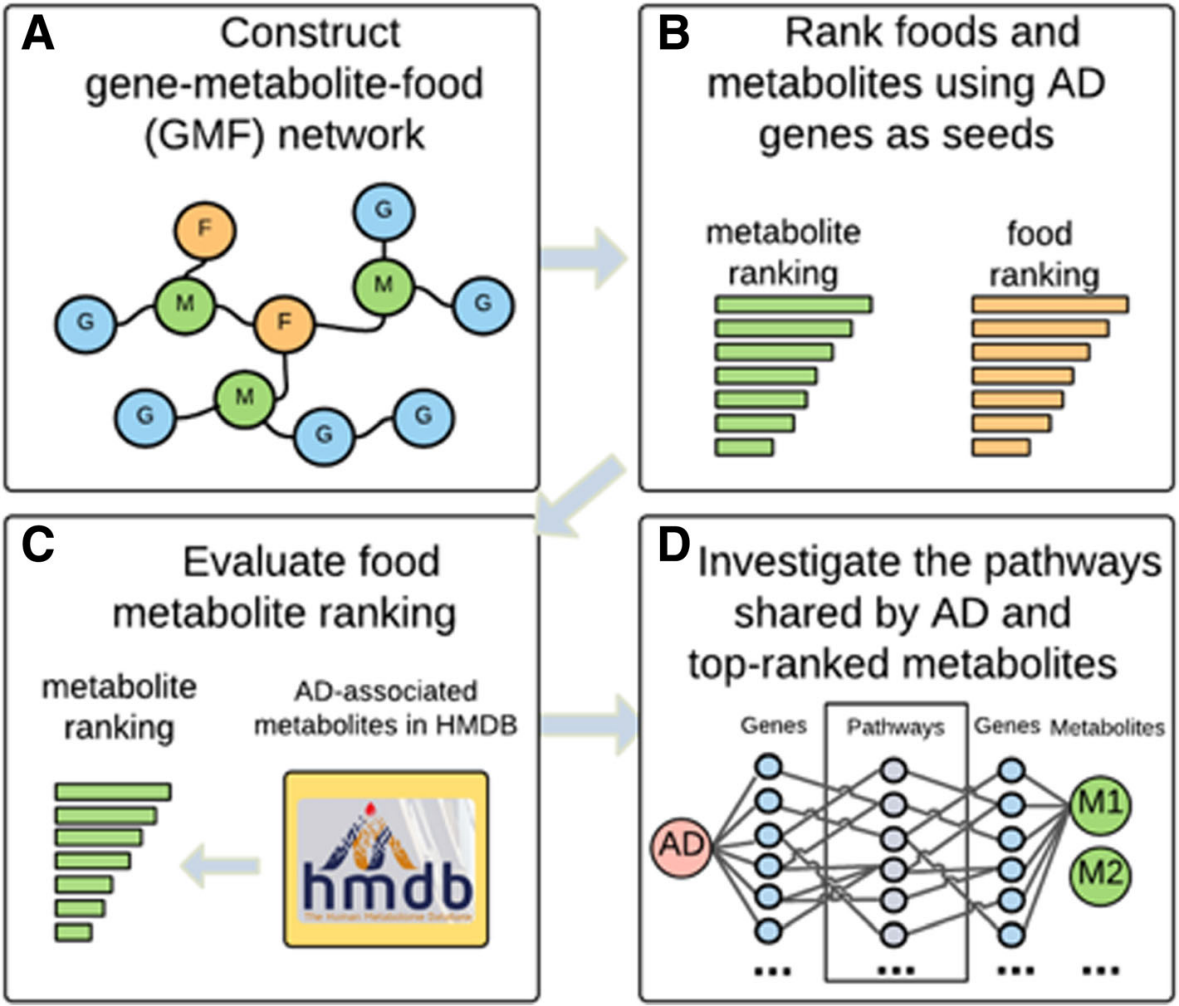
Four steps of our study: (1) GMF network construction (blue nodes: genes; green nodes: metabolites; orange nodes: food); (2) metabolite ranking using a network-based ranking algorithm; (3) evaluation of the metabolite ranking; and (4) investigation of the common pathways between AD and prioritized food metabolites

Our study consists of four steps (Fig. 2): first, we construct the GMF network using databases in Fig. 1; second, we prioritize AD-associated metabolites using a network-based ranking algorithm with the input of AD-causing genes; third, we evaluate the metabolite ranking using the known disorder-metabolite associations provided by HMDB; and finally, we investigate the common pathways shared by AD and top-ranked food metabolites to gain insights into how the metabolites affect AD. The following subsections describe each step in details.

### GMF network construction

We construct a context-sensitive network to model the interconnections among foods, metabolites, and human genes. We first extract the three types of nodes for the network: the metabolite nodes are extracted from HMDB [16]; the gene nodes are obtained from The HUGO Gene Nomenclature Committee (HGNC) [30] and labeled by approved gene names. For food nodes, we extract food names from FooDB [17] and normalize these strings using the unique identifier assigned by the database. Then we use the “group” information provided in FooDB for each food to further clean the food names: we exclude the foods in the group of “dishes”, such as “pizza” and “meatball”, which contain complex and uncertain components, and remove the food names that are high level food group names, such as “herbs and spices”, “fruits”, and “green vegetables”.

**Fig. 3:**
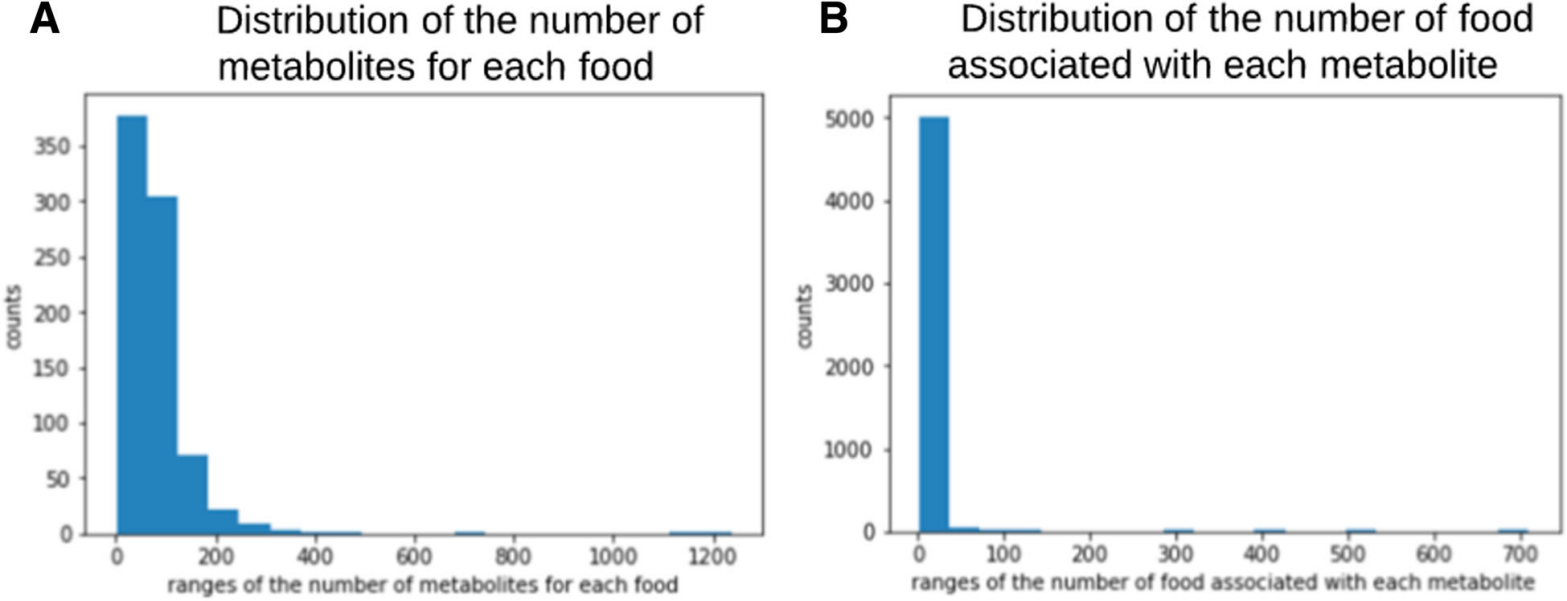
Distribution of (1) the number of metabolites for each food, and (2) the number of food associated with each metabolite

Next, we identify three types of edges for the network: metabolite-gene, metabolite-food, and gene-gene links. The metabolite-food edges are extracted from FooDB: we aligned the unique metabolite identifiers provided by FooDB to the metabolite names in HMDB. We conducted distribution analysis on food metabolites (Fig. 3). Each food is averagely associated with 78 metabolites, and 95% of the metabolites are linked to less than 20 foods. The metabolite-gene connections are extracted from the STITCH^18^ database: we link the metabolite names to PubChem compound identifiers, which is linked to interacting genes in STITCH. In addition, genes are connected other gene nodes via the protein-protein interactions extracted from the Search Tool for the Retrieval of Interacting Genes/Proteins (STRING) [31]. Since protein-protein interactions in STRING and metabolite-gene interactions in STITCH have confidence scores provided by each own database, we establish weighted edges for gene-gene and gene-metabolite edges, and normalize the weights into the range of [0,1]. Table 1 shows the size of the entire GMF network, and the numbers of nodes and edges of each kind.

**Table 1:** Number of nodes and edges in the gene-metabolite-food (GMF) network

	Node/edge type	Number
Nodes	Gene nodes	18,338
	Metabolite nodes	7596
	Food nodes	790
	Total	26,724
Edges	Gene-gene	7,869,282
	Gene-metabolite	210,405
	Metabolite-food	62,216
	Total	8,141,903

### Metabolite ranking algorithm

We first extracted from the Online Mendelian Inheritance in Man (OMIM) database all 14 genes associated with AD [32], and set the corresponding gene nodes in the GMF network as the “seeds.” Then we rank the nodes in the GMF network using the random walk model, which assumes that a walker starts from the seeds and randomly jumps to the neighbor nodes. We calculate an iteratively updated score for each node as the probability of being reached by the seeds:

1 $$ {p}_i=\left(1-\gamma \right)M{p}_{i-1}+\gamma {p}_0, $$

where *M* is the transition matrix, *γ* is the probability of restarting from the seeds, and *p*_0_ consists of the initial scores for all nodes. Here, the initial score is 1/14 for each seed and zero for all other nodes; thus all the scores add up to 1. The transition matrix *M* is the adjacency matrix of the GMF network after column-wise normalization. We set the restarting probability *γ* as 0.7 and the algorithm is insensitive to different choices of *γ*. We assume the algorithm converges if the difference of scores between iteration *ε* < 10^−8^. After the algorithm converges, we extract metabolites from all the nodes and rank them based on the scores.

### Evaluation of metabolite ranking

HMDB provides metabolite-disorder associations curated from literature. We extract a total of 81 AD-associated metabolites from HMDB, 59 of which appear in STITCH with associated genes. We used these 56 metabolites as the evaluation set. Most of metabolite-AD associations were identified in previous animal model or human cell line studies. Here, though the 59 metabolites are not the perfect gold standard for AD-associated metabolites, we consider them as the positive examples that show relevance with AD and test if they tend to be ranked highly in our approach.

We calculate the mean and median ranks for the 59 metabolites among our ranking. We also plot the precision-recall curve, and calculated the average precision across all recall levels when considering top *k* retrieved metabolites as the positive. The evaluation metrics are compared between our approach and the random cases. Pure random rankings result in a mean average rank of 50% for the 59 metabolites. Here, we generate random rankings by randomly selecting the seeds on the GMF network. Comparing our ranking for the evaluation set with the randomized cases, we test if the top-ranked metabolites were prioritized by chance.

### Pathway analysis for top-ranked food metabolites

Only part of the 7596 metabolites are actually linked to food nodes based on the FooDB data in the GMF network. In addition, many of the food metabolites are components of hundreds of different foods. We first extract the metabolites that were uniquely identified in less than ten foods. Then we identify the significantly enriched pathways for each top-ranked food-specific metabolite: we import the metabolite interacting genes into the QIAGEN’s Ingenuity Pathway Analysis software (IPA®, QIAGEN Redwood City, https://www.qiagenbioinformatics.com/products/ingenuity-pathway-analysis/) and download the significant canonical pathways. To compare the pathways for prioritized metabolites and AD, we also identified significant pathways for AD using the 14 AD-associated genes from OMIM.

We developed a method to rank the common significant pathways between AD and each prioritized metabolite. Intuitively, we intended to prioritize the pathways that are highly enriched for both AD- and metabolite-associated genes. The IPA software provides a coverage score for each AD- or metabolite-associated significant pathway; the score measures the percentage of AD- or metabolite-associated genes in each pathway. We design a score for each common pathway between AD and a metabolite to ensure the balanced coverage:

2 $$ s=\frac{c_{AD}\times {c}_m}{c_{AD}+{c}_m}, $$

where *c*_*AD*_ and *c*_*m*_ are the coverage of AD-associated genes and the metabolite-associated genes, respectively. The score was inspired by the definition of F1 measure, which is a measure of a test’s accuracy, and considers precision and recall at the same time. Last, we examine the top-ranked common significant pathways between AD and each metabolite based on the balanced score.

## Results

### Metabolite ranking based on the context-sensitive GMF network are supported by existing knowledge

**Fig. 4:**
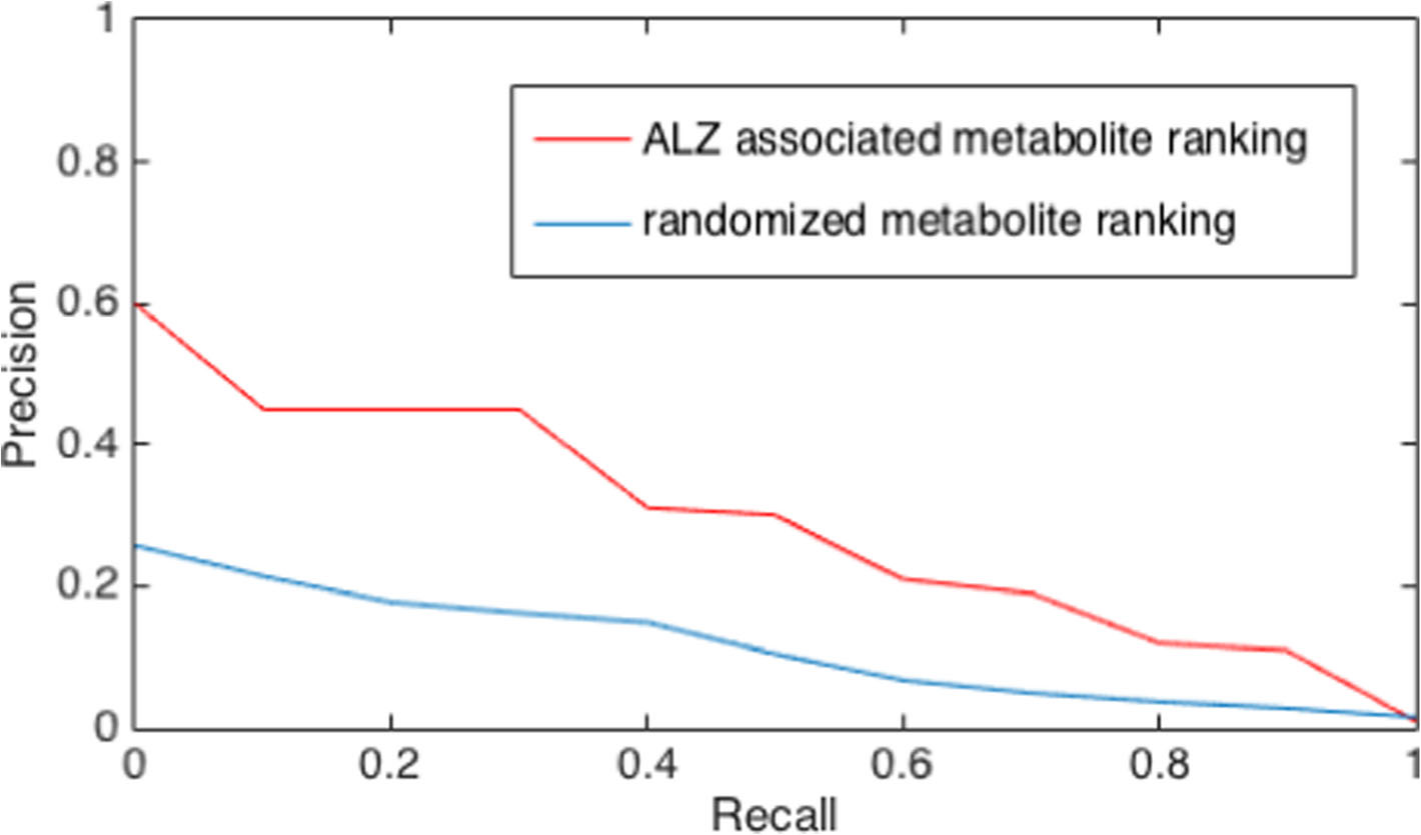
Precision recall curve for GMF network ranking algorithm for food-contained metabolites and the average of 100 random rankings

Our approach averagely ranked the 59 known AD-associated food metabolites in top 4.9% among the 5192 food metabolites in the GMF network (metabolite nodes that have connections to food nodes). Comparing with the randomized rankings (generated with random seeds placed on the GMF network), we achieved significantly higher mean rank (p < e-12, student’s T test) and median rank (p < e-14, Wilcoxon ranked sum test). Also, 55 out of the 59 (93%) positive examples of AD-associated metabolites were ranked within top 10%. In addition, the precision-recall curve in Fig. 4 demonstrates a better performance of our ranking comparing with the randomized rankings; the mean average precision calculated from the precision-recall curve is also significantly higher than the random case (Table 2, p < e-8). Together, the results demonstrate that our ranking for the food metabolites was able to prioritize relevant compounds for AD. Note that our ranking algorithm is unbiased, and did not use any prior knowledge about the known AD-associated food metabolites.

**Table 2:** Performance of metabolite ranking using the reduced GMF network comparing with the average performance of random rankings

Ranking	Mean rank	Median rank	Mean average precision
GMF network ranking	4.9%	1.9%	0.287
Randomized ranking	11.4%	18.2%	0.093

**Fig. 5:**
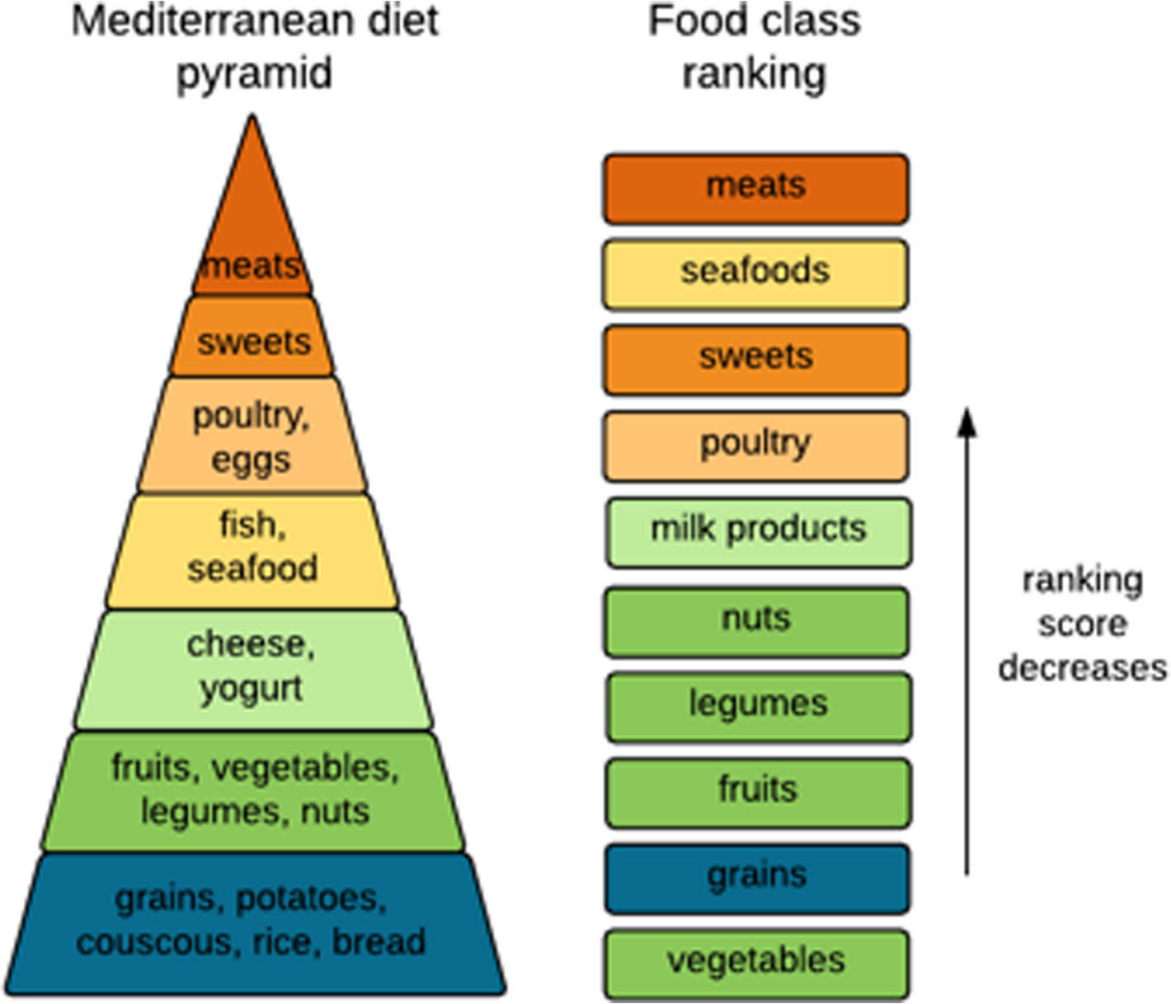
Mediterranean diet pyramid and food category ranking based on the GMF network

Besides the ranking for metabolites, our approach also automatically generated the ranking for all foods based on the strength of their associations with AD. We grouped the foods into categories and ranked the categories based on the average of food ranks in each category. The ranking shows a trend that high-fiber foods, such as grains, vegetables and legumes, tend to have higher scores than meats, sweets and milk products. Interestingly, our ranking is approximately correlated with the Mediterranean diet pyramid, which suggests an eating pattern with many healthy grains, fruits, vegetables, beans and nuts, and small amounts of dairy, red wine and meats [33] (Fig. 5). Here, the ranking of food categories only reflects the average ranks for foods of each class, and individual food in lowly ranked food categories may also contain metabolites that are closely relevant to AD. Next, we specifically examined each top-ranked food metabolites.

### Top-ranked food metabolites contain interesting candidates of AD-associated compounds

Many top-ranked metabolites are common nutrients found in hundreds of different foods, such as calcium and glycerol. Here, we focus on the unique metabolites that were exclusively identified in several specific foods or food categories. Table 3 lists the top-ranked food metabolites that were identified in less than ten foods. Seven out of ten metabolites were constituents of “healthy foods,” which include fruits, vegetables, grains, nuts and legumes. Among them, tetramethylpyrazine has been shown to exhibit the neuroprotective effects in rats [34]; and resveratrol is widely-known nutritional supplement with a number of beneficial health effects, such as anti-cancer [35], antiviral [36], neuroprotective [37–40], anti-aging [41], anti-inflammatory [42], cardioprotective [43], and life-prolonging effects. Among the top ten food-specific metabolites, only 4-hydroxynonenal was in the evaluation set of food metabolites that were known to be associated with AD based on the HMDB data. This also shows that many AD-associated food metabolites may not be readily included in existing databases. The ultimate goal of our study is to identify these new relevant food metabolites, which may might shed lights on the disease prevention.

**Table 3:** Top-ranked unique metabolites that were found in less than ten foods

Metabolite	Food group	Rank among all
estradiol	fruits, legumes	0.12%
tetramethylpyrazine	fruits, vegetables	0.18%
resveratrol	fruits, nuts	0.22%
theophylline	fruits	0.46%
chloroform	herbs and spices	0.47%
4-hydroxynonenal	legumes	0.55%
capsaicin	herbs and spices	0.62%
chlorine	fruits, vegetables	0.68%
emodin	herbs and spices, vegetables	0.75%
xylene	nuts, grains	0.76%

Surprisingly, we found that three metabolites in Table 3 are uniquely identified in the group of “herbs and spices”. Previous studies point out that the incidence of neurodegenerative diseases among people living in the Asian subcontinent, where people regularly consume spices, is much lower than in countries of the western world [44]. In addition, both in vitro and in vivo studies have indicated that nutraceuticals derived from herbs and spices, such as red pepper, black pepper, ginger, garlic, and cinnamon, target inflammatory pathways, and may show effects in preventing neurodegenerative diseases [45, 46]. We filtered our metabolite ranking and systematically extracted the compounds that are specifically found in herbs and spices. Table 4 lists the top ten spice-specific metabolites. Among these chemicals, capsaicin has been studied in animal models to investigate if it may attenuate memory impairment [47, 48]. Next, we systematically investigated the pathways targeted by the top AD-associated spice-specific metabolites.

**Table 4:** Top-ranked herbs and spices specific metabolites

Metabolite	Food	Rank among all
chloroform	spearmint	0.6%
capsaicin	ginger, pepper (*C. frutescens*), pepper (*C. annuum*)	0.79%
2,6-di-tert-butyl-4-methylphenol	soft-necked garlic	1.16%
sesamol	sesame, fats and oils	1.89%
desmosterol	cardamom, soy bean	2.56%
santene	parsley, rosemary, cornmint	3.18%
1-piperidinecarboxaldehyde	herbs and spices, pepper (spice)	3.28%
p-menthan-3-ol	herbs and spices	4.5%
sanguinarine	opium poppy	4.77%
1,1,1,3,3,3-hexachloro-2-propanone	herbs and spices	5.26%

### Top-ranked spice-specific metabolites share significant pathways with AD

**Fig. 6:**
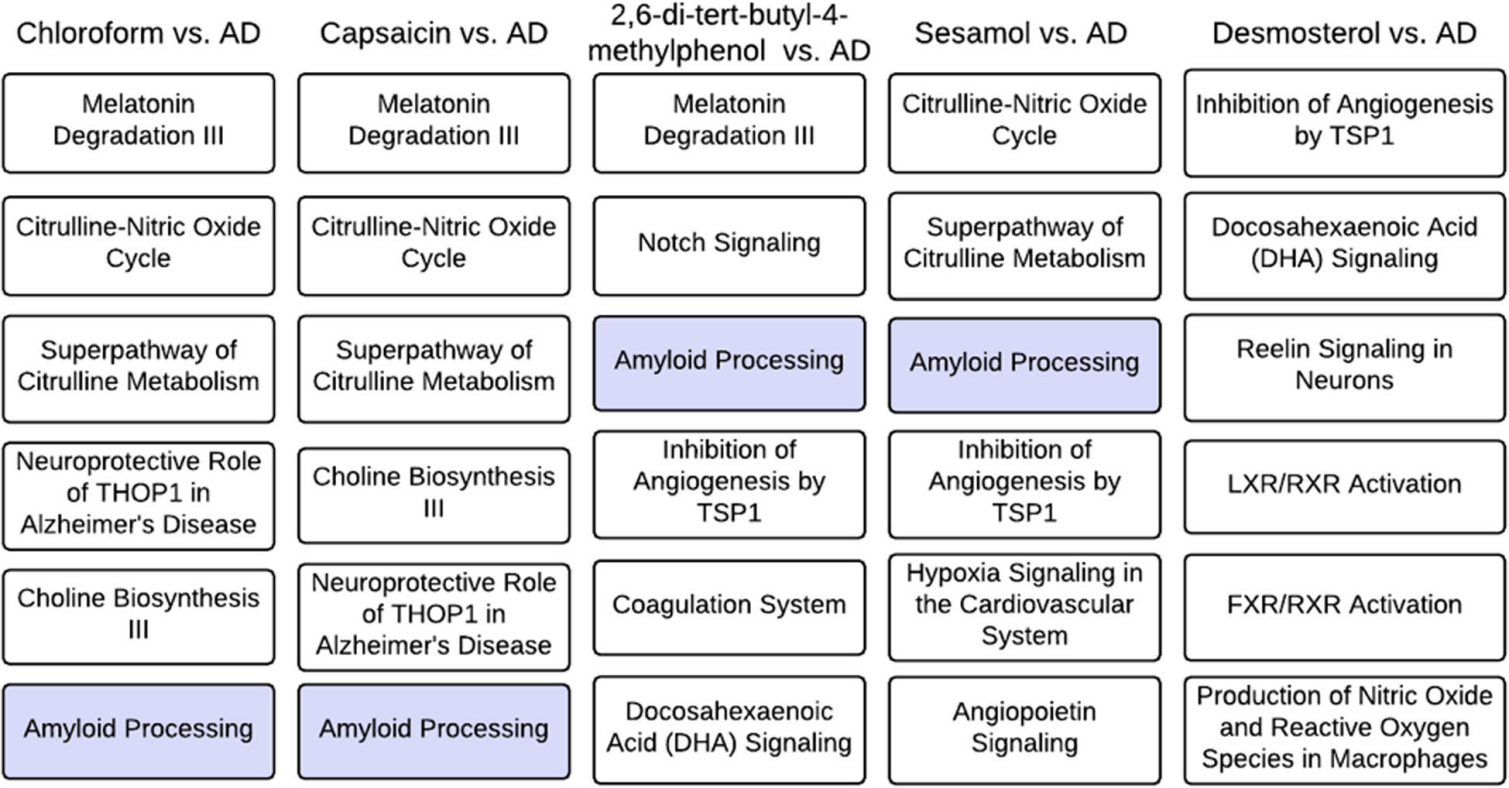
Overlapping pathways between the top-ranked herbs and spices specific metabolites and AD

We identified 58 significantly enriched pathways for AD, and found that each top-ranked herb-and-spice metabolite has many overlapping pathways with AD. Figure 5 shows the overlapping pathways that are mostly enriched for both AD- and metabolite-associated genes. Importantly, we found that amyloid processing (highlighted in Fig. 6) appears repetitively among the enriched pathways for herb-and-spice metabolites. The accumulation of the beta-amyloid protein is a major neuropathological hallmark in AD-affected brains and has a pathological role in AD [49]. The pathway analysis supports that the identified herb-and-spice metabolites are potentially involved with the development of AD. Other AD-involved pathways, including melatonin degradation [50], neuroprotective role of THOP1 [51], and Reelin signaling in neurons [52], were also found enriched for the herb-and-spice metabolite interacting genes. As a control, we also investigated the pathways for guanosine 2′,3′-cyclic phosphate, which is food metabolite ranked in the bottom by our approach; the metabolite has no overlapping pathways with AD.

## Discussions

We developed a novel context-sensitive network approach to analyze interactions among food, food metabolites, host genetics and pathways in the context of specific diseases. In this study, we use the approach to identify relevant food metabolites for AD, which is a complex disease affected by both genetic and environmental factors. Our study provides intriguing evidence for the role of diet, as an important environmental factor, in AD etiology. We also provide the hypotheses for the subsequent biological and clinical studies of host-environment interactions in AD. Due to the lack of gold standard (i.e., known food metabolites for many diseases), we did not test our algorithm on all other diseases. Our approach is not biased towards to AD; it is highly generic and can be applied to any other diseases.

The future work of this study includes the following aspects. (1) We will test and apply the algorithms to other food-related diseases, such as cancers, inflammatory bowel diseases, and allergy. (2) We will further classify food metabolites into neuroprotective and neurotoxic. In the future, as more detailed and quantitative data become increasingly available, we will be able to further classify the effects of food metabolites into AD-promoting or protective. (3) We constructed a network that contains gene, food, and metabolite nodes in this study. Other types of data, such as disease-phenotype relationships and disorder-metabolites in HMDB, may also be helpful in inferring AD-associated food metabolites. However, the usefulness of these data requires further evaluation. In the future, we will investigate effective approaches to rationally integrate more comprehensive data to predict AD-affecting food metabolites. (4) We will further improve the prediction algorithm based on the context-sensitive networks. In social network analysis, researchers have developed improved random walk algorithms that consider the semantic meanings of the paths in networks [53]. However, these approaches usually require prior knowledge or sufficient training data, to define or learning meaningful paths for the random walker in the network; the knowledge and training data cannot be easily obtained in most biomedical prediction scenarios. We will explore new algorithms in the unsupervised fashion that could further take the advantages of the context-sensitive networks. (5) In addition, we need further validation on the prioritized AD-associated food metabolites and how they might affect AD. Currently, we investigated the common significantly enriched pathways between AD and the prioritized metabolites, and found that a few metabolites are involved in the amyloid processing pathways. Amyloid processing is a major activity in AD-affected brains and involves with the cause of AD. The result shows that the top-ranked food metabolites are highly associated with AD development. However, further validations are essential through in vitro and in vivo experiments (6) Finally, AD may be related with the interactions of different food metabolites. More generally, other environmental factors, including toxins, drugs, and gut microbiome may also contribute to the AD development. In our previous work, we have studied brain-gut-microbiome connections in AD [54]. In the future, we will develop approaches in identifying chemicals from other sources that are associated with AD. We will also explore more complex computational models to investigate the combined effects of multiple environmental factors.

## Conclusions

In summary, we developed a novel network-based approach to understanding how food and food-derived metabolites are involved in complex human diseases, and conducted an exploratory study in AD. The identification of disease-associated food metabolites and their underlying pathways may provide insights into disease mechanism and offer the opportunities for disease prevention and treatment.

## Abbreviations

AD: Alzheimer’s disease
FooDB: The Food Database
GMF: Gene-metabolite-food
HMDB: The Human Metabolome Database
OMIM: The Online Mendelian Inheritance in Man
STITCH: Search Tool for Interactions of Chemicals
STRING: The Search Tool for the Retrieval of Interacting Genes/Proteins
